# Supplementary material for: Gamete expression of TALE class HD genes activates the diploid sporophyte program in Marchantia polymorpha
Source: eLife. 2021 Sep 17;10:e57088. doi: 10.7554/eLife.57088 (PMC8476127; doi:10.7554/eLife.57088)
Supplement: Figure 1—source data 1. [file elife-57088-fig1-data1.pdf]

Figure 1—source data 1. Molecular lesions of alleles listed in Figure 1 and Table 1.

Single knock-out lines

```
                                        gRNA1       PAM                                                  gRNA2       PAM
wt MpKNOX1     5'- - GACACCACTTCAGAACACAGCTGATGGTAAACAGGATGTGACCCT NNNN AAGCAGAGCGAAAGTGATTCGGTCAGTGGTGGGCAAAGTGAG - - 3'
Mpknox1-6      5'- - CAGACTTGGAGACACCACTTCAGAACACAGCTGATGGTA- - - - - - - - - - - - - - - - - - - - TGGTGGGCAAAGTGAG - - 3' -2359 bp female
```

```
                                      gRNA1       PAM
wt MpBELL2     5'- - CAATGGATTATGAAGCTCGTCACTCAC AAGAGGTGCTTTCGCCTGTTCCA - - 3'
Mpbell2-1      5'- - CAATGGATTATGAAGCTCGTCACTCACcAAGAGGTGCTTTCGCCTGTTCCA - - 3'        +1 bp female
Mpbell2-2      5'- - CAATGGATTATGAAGCTCGTCACT - - - - - GCTTTCGCCTGTTCCA - - 3'       -10 bp male
Mpbell2-11     5'- - CAATGGATTATGAAGCTCGTCACTCA- AAGAGGTGCTTTCGCCTGTTCCA - - 3'        -1 bp male
```

```
                                      gRNA1       PAM                                                 gRNA2       PAM
wt MpBELL3     5'- - TATCCTAAAGGAGACGAGTGGGGGCCACTTGTGGCTTCCACAGG NNNN ACAACGAGCGCCCATTCCCCCAGGCTGAACAGTACGGGGTCACC - - 3'
Mpbell3-4      5'- - TATCCTAAAGGAGACGAGTGGGGGCCAC - - - - - - - - - - - - - - - - - - - - - - - - -  AGTACGGGGTCACC - - 3' -2217 bp male
Mpbell3-10     5'- - TATCCTAAAGGAGACGAGTGGGGGCCAC - - - - - - - - - - - - - - - - - - - - - - - - - - GTACGGGGTCACC - - 3' -2218 bp male
```

```
                               PAM        gRNA1                                               gRNA2       PAM
wt MpBELL4     5'- - AGAACGAATTGCACCACGACAGCCTCCTTGGGTTCAGGAAGTTGGAACT NNNN TTTCTCGTAATCGGAGGAACAACAATGGGAATCGTAATA - - 3'
Mpbell4-5      5'- - AGAACGAATTGCACCACGACA - - - - - - - - - - - - - - - - - - - - - - - - - - - - AATGGGAATCGTAATA - - 3' -1135 bp male
Mpbell4-24     5'- - AGAACGAATTGCACCACGACA - - - - - - - - - - - - - - - - - - - - - - - - - - - - AATGGGAATCGTAATA - - 3' -1135 bp male
```

Multiple gene knock-out lines

***Mpbell34-4* (*Mpbell3-1 Mpbell4-1*) male**

```
                                       gRNA1          PAM
wt MpBELL3     5'- - TATCCTAAAGGAGACGAGTGGGGGCCA       CTTGTGGCTTCCACAGGAATGCAAAACAA - - 3'
Mpbell3-1      5'- - TATCCTAAAGGAGACGAGTGGGGGCCAtctcct-TTGTGGCTTCCACAGGAATGCAAAACAA - - 3' +6 bp/-1 bp

                                         gRNA2        PAM
wt MpBELL3     5'- - TGACAACGAGCGCCCATTCCCCCAGGCTGAACA GTACGGGGTCACC - - 3'
Mpbell3-1      5'- - TGACAACGAGCGCCCATTCCCCCAGGCTGAACAaGTACGGGGTCACC - - 3'  +1 bp

                               PAM        gRNA1
wt MpBELL4     5'- - AGAACGAATTGCACCACGACAGCCTCCTTGGGTTCAGGAAGTTGGAACT - - 3'
Mpbell4-1      5'- - AGAACGAATTGCACCACGA-AGCCTCCTTGGGTTCAGGAAGTTGGAACT - - 3'  -1 bp
```

***Mpbell234-15* (*Mpbell2-15 Mpbell3-15 Mpbell4-15*) male**

```
                                                gRNA1                     PAM
wt MpBELL2     5'- -  CAATGGATTATGAAGCTCGTCA                        CTCACAAGAGGTGCTTTCGCCTGTTCCA - - 3'
Mpbell2-15     5'- -  CAATGGATTATGAAGCTCGTCAagagcaagcacctctttgcttgc - - - - -GTGCTTTCGCCTGTTCCA - - 3' +23 bp/-10 bp
```

```
                                                                    gRNA1                                PAM
wt MpBELL3     5'- - TATCCTAAAGGAGACGAGTGGGGGCCA                                                   CTTGTGGCTTCCACAGGAATGCAAAACAA - - 3'
Mpbell3-15     5'- - TATCCTAAAGGAGACGAGTGGGGGCCAaaacaatgagtaagtgttcaaatacctgattgttttggagtggagacga-TTGTGGCTTCCACAGGAATGCAAAACAA - - 3' +49bp/-1bp

                                      gRNA2         PAM
wt MpBELL3     5'- - TGACAACGAGCGCCCATTCCCCCAGGCTGAACA GTACGGGGTCACC - - 3'
Mpbell3-15     5'- - TGACAACGAGCGCCCATTCCCCCAGGCTGAACAaGTACGGGGTCACC - - 3' +1 bp

                              PAM        gRNA1
wt MpBELL4     5'- - AGAACGAATTGCACCACGACAGCCTCCTTGGGTTCAGGAAGTTGGAACT - - 3'
Mpbell4-15     5'- - AGAACGAATTGCACCACGA-AGCCTCCTTGGGTTCAGGAAGTTGGAACT - - 3' -1 bp
```

***Mpbell234-83* (*Mpbell2-83 Mpbell3-83 Mpbell4-83*) male**

```
                                                            gRNA ATG      PAM                                gRNA2      PAM
wt MpBELL2     5'- - AACTTCTCGTCGAAGATGTATTT NN GGATTATGAAGCTCGTCACTCACAAGAGGTGCTTTC NN GCAAAGGGAAGATTTAGCAAAGGAATCTGGAGTTACCTATGT - - 3'
Mpbell2-83     5'- - AACTTCTCGTCGAAGATGTATTT- - - - - - - - - - - - - ggaggc-110bp-atatac- - - - - - - - - - - - - -ATCTGGAGTTACCTATGT - - 3' +110
bp/-1401 bp

                                     gRNA1        PAM                                          gRNA2       PAM
wt MpBELL3    5'- - TATCCTAAAGGAGACGAGTGGGGGCCACTTGTGGCTTCCACAGG NNNN ACAACGAGCGCCCATTCCCCCAGGCTGAACAGTACGGGGTCACC - - 3'
Mpbell3-83    5'- - TATCCTAAAGGAGACGAGTGGGGGCCAC - - - - - - - - - - - - - - - - - - - - - - - - -  AGTACGGGGTCACC - - 3' -2217 bp

                             PAM       gRNA1                                            gRNA2       PAM
wt MpBELL4    5'- - AGAACGAATTGCACCACGACAGCCTCCTTGGGTTCAGGAAGTTGGAACT NNNN TTTCTCGTAATCGGAGGAACAACAATGGGAATCGTAATA - - 3'
Mpbell4-83    5'- - AGAACGAATTGCACCACGA - - - - - - - - - - - - - - attgcac - - - - - - - - - - - AATGGGAATCGTAATA - - 3' +7 bp/-1135 bp
```

***Mpbell234-7* (*Mpbell2-7 Mpbell3-7 Mpbell4-7*) female**

```
                                          gRNA1                    PAM
wt MpBELL2     5'- - CAATGGATTATGAAGCTCGTCACTCAC                     AAGAGGTGCTTTCGCCTGTTCCA - - 3'
Mpbell2-7      5'- - CAATGGATTATGAAGCTCGTCACTCAC - - gacggtgaaagcacct - - - CTTTCGCCTGTTCCA - - 3' +16 bp/-8 bp

                                     gRNA1       PAM                                                      gRNA2       PAM
wt MpBELL3     5'- - TATCCTAAAGGAGACGAGTGGGGGCCACTTGTGGCTTCCACAGGAATGCAAAACAATCA NNNN CGAGCGCCCATTCCCCCAGGCTGAACAGTACGGGGTCACC - - 3'
Mpbell3-7      5'- - TATCCTAAAGGAGACGAGTGGG - - - - - - - - t - - - - - - - -ACAATCA NNNN CGAGCGCCCATTCCCCCAGGCTGAACAGTACGGGGTCACC - - 3' +1
bp/-30bp ; wt

                              PAM                         gRNA1
wt MpBELL4     5'- - AGAACGAATTGCACCACGA                                             CAGCCTCCTTGGGTTCAGGAAGTTGGAACT - - 3'
Mpbell4-7      5'- - AGAACGAATTGCACCACGAcgaattcgttatacacataaaaaaaattatgttattcgaatacaCAGCCTCCTTGGGTTCAGGAAGTTGGAACT - - 3'  +44 bp
```

Mp*bell234-18* (Mp*bell2-18* Mp*bell3-18* Mp*bell4-18*) female

gRNA1 PAM
wt Mp*BELL2* 5'– – CAATGGATTATGAAGCTCGT CACTCACAAGAGGTGCTTTCGCCTGTTCCA – – 3'
Mp*bell2-18* 5'– – CAATGGATTATGAAGCTCGT – – gagtgacgaggtg – – – – AAGAGGTGCTTTCGCCTGTTCCA – – 3' +13 bp/-6 bp

gRNA1 PAM gRNA2 PAM
wt Mp*BELL3* 5'– – TATCCTAAAGGAGACGAGTGGGGGCCACTTGTGGCTTCCACAGGAATGC NN GTAAATCGAAT NN CGAGCGCCCATTCCCCCAGGCTGAACA GTACGGGGTCACC – – 3'
Mp*bell3-18* 5'– – TATCCTAAAGGAGACGAGTGGG- – – – atccaa-97bp-cgatcc – – TAAATCGAAT NN CGAGCGCCCATTCCCCCAGGCTGAACAtGTACGGGGTCACC – – 3' +97 bp/-304; +1 bp

PAM gRNA1
wt Mp*BELL4* 5'– – AGAACGAATTGCACCACGACAGCCTCCTTGGGTTCAGGAAGTTGGAACT – – 3'
Mp*bell4-18* 5'– – AGAACGAATTGCACCACGA-AGCCTCCTTGGGTTCAGGAAGTTGGAACT – – 3' -1 bp
